# Indoor Air Quality Improvement Using Nature-Based Solutions: Design Proposals to Greener Cities

**DOI:** 10.3390/ijerph18168472

**Published:** 2021-08-11

**Authors:** Teresa M. Mata, Gisela M. Oliveira, Helena Monteiro, Gabriela Ventura Silva, Nídia S. Caetano, António A. Martins

**Affiliations:** 1 INEGI-Institute of Science and Innovation in Mechanical and Industrial Engineering, R. Dr. Roberto Frias 400, 4200-465 Porto, Portugal; gventura@inegi.up.pt; 2 UFP Energy, Environment and Health Research Unit, University Fernando Pessoa, Praça Nove de Abril, 349, 4249-004 Porto, Portugal; gisela@ufp.edu.pt; 3 Low Carbon & Resource Efficiency, R&Di, Instituto de Soldadura e Qualidade, 4415-491 Grijó, Portugal; himonteiro@isq.pt; 4 LAETA-Associated Laboratory for Energy and Aeronautics, R. Dr. Roberto Frias, S/N, 4200-465 Porto, Portugal; 5 LEPABE-Laboratory for Process Engineering, Environment, Biotechnology and Energy, Faculty of Engineering, University of Porto (FEUP), R. Dr. Roberto Frias, S/N, 4200-465 Porto, Portugal; nsc@isep.ipp.pt (N.S.C.); amartins@fe.up.pt (A.A.M.); 6 CIETI, Department of Chemical Engineering, School of Engineering (ISEP), Polytechnic of Porto (P.Porto), R. Dr. Antonio Bernardino de Almeida 431, 4249-015 Porto, Portugal

**Keywords:** CO_2_ mitigation, indoor air quality, microalgae, health impact mitigation, nature-based solutions, circular economy

## Abstract

Low indoor air quality is an increasingly important problem due to the spread of urbanization. Because people spend most of their time inside, poor indoor air quality causes serious human health issues, resulting in significant economic losses. In this work, the current state of affairs is presented and analyzed, focusing on the current problems and the available solutions to improve the quality of indoor air, and the use of nature-based solutions. These involve the cultivation of microalgae in closed photobioreactors. In these systems, photosynthetic organisms can capture CO_2_ and other pollutants generated in indoor environments, which they use to grow and develop biomass. Several possible layouts for the implementation of microalgae-based indoor air cleaning systems are presented, taking into account the systems that are currently available at a commercial scale. A critical analysis of the microalgae indoor purification systems is presented, highlighting their advantages and disadvantages, and suggesting potential improvements and future lines of research and development in the area.

## 1. Introduction

Climate change has affected the economies and societies of all countries, and this trend of global effects is expected to continue for some decades, despite the increase in activities to mitigate greenhouse gas (GHG) emissions into the atmosphere. Emissions of carbon dioxide (CO_2_) and other GHGs decreased by around 6% in 2020 due to travel bans and the economic slowdown resulting from the COVID-19 pandemic. However, this improvement was only temporary, as recovery in the global economy led to emissions returning to, or surpassing, their previous levels, and the exacerbation of air pollution [1]. To address the climate emergency, countries’ post-pandemic recovery plans to rebuild their economies must trigger long-term systemic changes that will change the trajectory of CO_2_ and other GHGs in the atmosphere. These must shape economies to be more environmentally friendly, healthy, safe, and resilient. Thus, the current crisis could be an opportunity for a deep and systemic shift towards a more sustainable economy that is more beneficial for people and the planet. This will involve developing positive actions for the climate, such as decarbonizing the economy and investing in sustainable solutions, particularly those that are inspired by nature.

Sustainable development measures are being considered in various human activity sectors, aiming to curb GHG emissions and to generally improve environmental conditions by reducing energy consumption. The European Commission is working on a Zero Pollution Action Plan, as part of the “European Green Deal”, and reviewing its air pollution legislation, including the Ambient Air Quality Directives [2]. In addition, the 17 United Nations Sustainable Development Goals address the global development challenges, and represent a blueprint for achieving a better and more sustainable future. In particular, Goal 3 aims to ensure healthy lives and to promote well-being at all ages, which is essential to sustainable development; Goal 11 aims to make cities inclusive, safe, resilient, and sustainable; and Goal 13 aims to take urgent action to combat climate change and its impacts [3].

In the European Union (EU), almost 50% of the final energy consumption is used for heating and cooling, of which 80% is used in buildings, according to the Directive 2018/844/EU [4]. Specifically, for public services’ buildings, energy consumption is intended to be strongly reduced, and is one of the top targets of the *European Green Deal* strategy to become carbon neutral by 2050 [5]. To achieve such bold targets, European countries need to ensure that, in 2030, at least 32% of the EU’s energy share comes from renewable sources [6]. In addition, the EU established a legislative framework concerning the energy performance of buildings to improve their efficiency, and hoping to promote recommendations so that all new buildings, particularly new public buildings, will be Nearly Zero-Energy Buildings (NZEBs). This regulatory context includes the Energy Performance of Buildings Directive 2010/31/EU (EPBD) and the Energy Efficiency Directive 2012/27/EU, amended in 2018 [4]. According to these, an NZEB is a sustainable building with very low demand for energy, which must come mostly from renewable energy produced on site or nearby. However, for better energy performance, a set of active and passive measures needs to be implemented regarding buildings’ needs [7] in such a manner that does not compromise their functions [8]. Passive measures were the first to be developed, and benefit from construction design and the application of more thermal and energy-efficient materials for construction. These passive measures intend to reduce or eliminate the need for mechanical cooling, heating, and ventilation, and to improve the efficiency of lighting devices. One example of passive heating solutions for buildings is the “Trombe Wall”, one of the earliest well-known bioclimatic strategies [9].

Therefore, to develop sustainable buildings and cities with less pollution, one of the critical issues is to introduce renewable energy sources and to use energy storage with an integrated smart grid system that can supply and distribute energy equally and on demand. Regarding building construction and architecture, incorporation of solar energy has increased for thermal and electric purposes. As a renewable energy source, solar energy efficiency is highly dependent on the weather and requires a large area of land or surface available for the installation of solar collectors for large-scale solar energy production. Additionally, the energy requirement of some buildings (e.g., for heating, cooling, lighting, and electric appliances) is usually much greater than that which can potentially be generated by the commercially available solar systems. One possibility of benefiting from solar energy in buildings is to use it as a chemical source, in the same manner as photosynthetic organisms, by capturing solar light and transforming it into biomass stored in plants, trees, or microalgae. Several projects have presented green façade solutions that not only contribute to the energy efficiency of the buildings, but also improve their thermal comfort and aesthetic appeal.

Bayoumi and Fink [10] note that an energy-generating façade on a building cannot respond to its total energy need while maintaining the visual comfort of the occupants. This means that, despite the technological advances in photovoltaic cells, and even if it is located in a high sun exposure area, a building typically has a negative energy balance. The energy requirement for cooling a room is generally affected by both the external level of solar irradiation and the internal heat radiation from construction materials, humans, equipment, and lighting. The need for electric lighting is also affected by the availability of daylight, and the existence and type of windows and shading devices. It is difficult to quantitatively assess the thermal and aesthetic comfort of transparent façades, which are current fashionable. Previous research [10] presented a method for determining the optimum façade criteria for considerably enhancing building performance, addressing basic issues of energy balance (i.e., energy yield versus demand, measured in kWh m^−2^ year^−1^), user comfort, and the impact of the chosen criteria on the formal quality of the building. The simulation results show that the energy balance of the building per square meter clearly decreases exponentially as the number of floors increases. Therefore, this model shows that it will be difficult for high-rise buildings, with several floors, to be autonomous in terms of energy.

Due to their design and construction, new energy-efficient buildings are usually well-insulated and with increased airtightness, which considerably diminishes natural ventilation. In airtight buildings, the interior air quality may quickly degrade with high occupation levels, and mechanical ventilation systems are relied on to condition the air, usually with considerable thermal losses. Given the challenge of jointly improving energy efficiency and maintaining indoor air quality, the search for new technologies for local air purification is even more desirable. This paper aims to explore the possible nature-based solutions, using microalgae systems in buildings, as a means to improve air quality, capture indoor carbon dioxide, produce oxygen, biomass, and bioenergy, and improve buildings’ global performance. The potential benefits and drawbacks of these new types of systems are analyzed in this work.

## 2. Air Pollutants, Sources and Human Health Effects

Humans require air to breath and live, mainly via the supply of oxygen, O_2_, for cellular respiration. This provides the energy to operate the cellular metabolism in order to move, think, and perform other activities. Considering an adult respiratory rate of 12 to 18 breaths/minute, which equates to inhaling about half a liter of air, a person breathes approximately 17 m^3^ air/day [11]. Air quality and human health are intertwined, and air quality is widely recognized as being one of the most important causes of disease in the 21st century [12,13,14]. Although indoor air quality has received less attention than outdoor air pollution, in the presence of indoor sources, indoor contaminant concentrations are higher, and sometimes 10-fold higher than the respective outdoor air levels (e.g., formaldehyde, whose sources vary from furniture to cleaning agents). Furthermore, in contemporary European lifestyles, citizens spend, on average, over 90% of their time inside. The combination of the generally higher indoor concentration of certain pollutants, and the fraction of time spent inside, results in the overall domination of indoor air pollution exposure. In addition, each year, more than 5 million people die prematurely from diseases attributable to poor indoor air quality, which also contributes to high economic losses due to reduced worker productivity, increased health care costs, and other material losses [12]. Indoor air pollutants are diverse, ranging from particulate materials (PMs), biological pollutants, and over 400 different organic and inorganic chemical compounds, whose concentrations depend on both internal and external factors [15]. Any contaminants, either chemical and biological, can result in significant health problems, which may lead to temporary or even permanent incapacitation, and, in extreme cases, to death, especially of those more vulnerable, i.e., children and the elderly. For example, although only CO_2_ concentrations above 5% are considered to pose irreversible risks to health [16], a person is unable to breathe air in an atmosphere with a 4% CO_2_ concentration for more than several minutes without feeling sick. Recent data [17] suggests that chronic exposure to CO_2_ values of 1000 ppm may affect cognitive performance and contribute to increased symptoms of respiratory diseases. Air is also the media by which a person can be contaminated with several diseases (the airborne route of infection), including, for example, flu, tuberculosis, and now, the ongoing COVID-19 pandemic. In an urban environment, there are a large number of opportunities for disease transmission because people spend their time in closed environments: at their homes, on public transport, and in offices, classrooms, restaurants, shops, and theatres. Other examples of contaminants associated with poor indoor air quality are radon, which is linked to some types of cancer, tobacco smoke, and emissions of volatile organic compounds (VOCs) [18]. Table 1 presents examples of volatile and semivolatile organic compounds and inorganic pollutants normally found inside, their health effects, and their most probable sources.

Terpenes, compounds with a natural origin such as limonene, α-pinene, and β-pinene, are chemicals that may play an important role as indoor air pollutants. This is because they can be involved in oxidation reactions with ozone or nitrogen oxides that contribute to the formation of formaldehyde and ultrafine particles formed by condensation/nucleation processes as products of the reaction. The main sources of terpenes are cleaning products and disinfectants with terpene-based fragrances. Inhalation of terpenes is generally not considered to be a health concern but their reaction products may pose a concern [19].

Regarding nitrogen oxides, 90 to 95% are generally emitted as nitric oxide and only 5 to 10% as nitrogen dioxide, although large variations between sources have been observed. Under ambient conditions, nitric oxide is rapidly oxidized in air to form nitrogen dioxide by the available oxidants (such as oxygen, ozone, and VOCs), and this rapid oxidation rate is such that nitrogen dioxide is generally regarded as a primary pollutant [20].

Concerning carbon monoxide, inhalation is the only exogenous exposure route. Carbon monoxide is produced inside by combustion sources (cooking, heating, candles, incense, and smoking) and it is also introduced through the infiltration of carbon monoxide from outdoor air into the indoor environment. From outside, carbon monoxide can come from busy roads because it is emitted from the exhaust fumes of gasoline and diesel vehicles. Parking areas or private garages can also be a source of carbon monoxide [20].

Many sources of air contamination exist, both natural and anthropogenic. This work focuses on the later, and mainly on the consequences to the quality of indoor air in urban environments. In these environments, the outdoor air quality is usually degraded by higher population density, traffic, services, commerce, industry, and the other activities that comprise the urban setting [20,21]. If the outdoor air quality is poor, it is relatively difficult to refresh the indoor air to meet quality standards using traditional ventilation systems. Therefore, buildings’ systems to improve indoor air quality rely on several ventilation systems, with or without purification (filtration devices). Traditionally, this involves the use of an AHU (Air Handling Unit) or simply an AVAC (Air Ventilation and Air Conditioning System), whose most important function is to provide thermal comfort for buildings’ residents and users. Due to the COVID-19 threat of airborne disease transmission, at a scale not previously experienced by humanity, this solely thermal comfort perspective has been required to change for safety reasons. Increasing human development and standards of living have led to individuals spending an increasing amount of time inside, either at home (cooking, energy generation) or other indoor facilities, such as theaters/cinemas, work (e.g., offices, industrial units), schools, and hospitals. Each poses its own specific set of challenges, because different contaminants are generated, thus requiring different mitigation and/or air treatment approaches. A relevant example is the intensive use of indoor spaces, particularly in public buildings such as higher education institutions, which often have crowded classrooms, laboratories, libraries, or cafeterias. Presently, the safety of air, which relates to the microbiological and chemical compliance with international standards [20,22,23], has greater importance than the thermal comfort for which the NZEB and the energy efficiency EU Directives were designed. In an urban context, all of these complex problems have interrelated dimensions. The trend of increasing global average atmospheric temperatures is expected to continue in the coming decades, causing extreme heat events that are likely to become more frequent and severe in many cities, especially in the south of Europe and in the Mediterranean region. Additionally, this climate change impact is expected to worsen the urban heat island effect, which is characteristic of cities due to land use change, and the resulting increase in imperviousness and decrease in surface albedo, which increases radiation heat from the built environment [24,25].

This work aims to explore the possible nature-based solutions using microalgae systems, to alleviate the urban heat island effect while improving the air quality in buildings. The aim of these systems is to mimic the role played by microalgae in the oceans, namely, providing oxygen and biofixing carbon dioxide from the atmosphere. This study proposes that systems should be installed in new and already existing buildings to regenerate the building air in an energy-conserving manner and provide autonomy to the building. The designs of these systems are presented in this paper.

The Portuguese legislation on indoor air quality states that indoor air cannot have a CO_2_ concentration higher than 1250 ppm and, concerning air renovation flow calculations, takes the atmospheric CO_2_ level as the reference considering an average concentration of 390 ppm [26]. However, the current CO_2_ atmospheric mean concentration is accepted to have exceeded 415 ppm with an increasing frequency. The literature has described assessments of the indoor air quality in schools in Portugal [27,28,29] and abroad [30,31] because it is a matter of public health. Results confirmed that schools need to improve the quality of their indoor air, particularly in buildings in which mechanical ventilation systems are not used [27,31]. Other studies evaluated the indoor air quality of higher education buildings, showing that CO_2_ levels may reach peak concentrations [32]. For example, measurements taken during teaching hours showed a mean CO_2_ concentration value of 1530 ppm, varying in a range from −24% to +31% [30]. This study also showed that in high-density traffic areas or near industrial activities, outdoor air pollutants affect indoor air quality, particularly by increasing the levels of PM_10_, PM_2.5_, total volatile organic compounds, benzene, and toluene.

The ongoing human-driven climate change has resulted in more frequent and intense heat events, which are aggravated by droughts and wildfires. This development has forced an increase in energy demand for refrigeration and air conditioning, in addition to maintaining pressure on the existing water resources, which are scarce in the south of Europe. Because cities are expected to absorb most of the forecasted population growth in the next several decades, mitigation and adaptation measures for extreme weather events and catastrophes are particularly critical in the urban landscape due to the complexity of urban metabolism [33,34]. In the European Union, the construction sector is responsible for about 40% of energy consumption and 36% of GHG emissions [4], and is one of the main sources of environmental pollution, mainly due to the excessive emissions associated with the processes of heating and cooling systems in buildings. Thus, a significant improvement is needed with regard to the design of new buildings with lower energy needs, supported by the implementation of renewable energy systems [8].

Aligned with the EU Strategy of “A Clean Planet for all” [35], Portugal has also made a formal commitment to be carbon neutral by 2050. In two activity sectors, energy transformation and energy use in buildings, reductions in CO_2_ emissions of over 80% are foreseen to meet the defined targets and curb climate change effects. According to the International Renewable Energy Agency [36], improvements in renewable energy use would significantly reduce CO_2_ emissions from heating and cooling services in buildings. Thus, there is a need to deeply invest in new technologies and renewable energy.

## 3. Indoor Air Quality Control

Recognizing the vital importance of indoor air quality, the World Health Organization (WHO) has defined quality guidelines for indoor environments [20], providing reference thresholds based on scientific evidence of the harmful consequences for human health of pollutant exposure [12,37].

The pollutants referenced in the guidelines include benzene, carbon monoxide, formaldehyde, naphthalene, nitrogen dioxide, polycyclic aromatic hydrocarbons, radon, trichloroethylene, and tetrachloroethylene. Carbon dioxide is not considered a pollutant but as an indicator of air quality in buildings related to human metabolism [38]. However, it is known that the increase in inhaled carbon dioxide increases pulmonary ventilation and, thus, carbon monoxide uptake. Therefore, a high concentration of CO_2_ can enhance the toxicity of other compounds to humans, so it is important that it is regulated.

In many situations, the indoor air quality can be ensured using outside clean air to replace polluted indoor air. The existing legislation, regulations, and/or guidelines are based on this assumption. Minimal air renewal rates, based on the overall room/building volume, for different activity sectors and occupancy rates, can be found in the applicable regulations [39,40]. However, indoor air quality is constrained not only by the renovation rate of fresh air from outdoors, but also by the quality of atmospheric air that is brought inside [41]. In densely populated cities or industrial regions, where atmospheric air quality is often very poor, these criteria and/or recommendations are not adequate. This situation also applies to other pollutants. It should also be considered that pollution is often a regional problem with local constraints and characteristics. Air renovation from poor quality outdoor air may raise other concerns, particularly due to the presence of pollutants such as particulate matter (PM_10_ and PM_2.5_) or nitrogen oxides (NO_x_), which are recognized to be harmful and associated with certain diseases [42]. Although cities and metropolitan areas are sources of economic growth, contributing to around 60% of global GDP, they are also responsible for around 70% of global carbon emissions and use over 60% of the resources [3]. Road traffic is the biggest source of urban ambient air pollution, accounting for 25% of the global levels of particulate matter, followed by unspecified sources of human origin (22%), domestic fuel burning (20%), natural dust and sea salt (18%), and industrial activities including power generation (15%) [43].

Nonetheless, a problem persists: if the quality of atmospheric air is not sufficient to ventilate indoor environments, how can the problem of the accumulation of substances in indoor air be solved?

Several possibilities exist and have been implemented in practice. There are three strategies to promote good indoor air quality: source control, ventilation, and air cleaning or purification. Source control is the smartest strategy because it avoids the problem at the source. However, in some cases, it is not able to be applied due to constraints related to construction materials or ongoing activities. Ventilation requires energy and implies emissions to the ambient air, at both the local and global scales. One of the emergent areas of indoor air quality is related to cleaning technologies. Various air treatment technologies can be used for control of contaminants. Conventional processes, such as sorption onto solid sorbents (for VOCs), filtration (for particulate matter, PM), and disinfection (for bioaerosols and microorganisms), are combined with advanced treatment processes, such as photocatalytic oxidation of VOCs, bipolar air ionization to agglomerate PM, and ultraviolet disinfection to inactivate bioaerosols [44]. Despite their high applicability, these processes have several disadvantages. For instance, for PM reduction, in the case of filtration, frequent replacement of filters is required and, in the case of electrostatic precipitation, a high risk of ozone generation exists. UV-photocatalytic oxidation appears to be a promising air cleaning technology. However, issues remain to be addressed before it can be used safely in buildings, such as generation of formaldehyde and acetaldehyde from partial oxidation of ubiquitous VOCs such as alcohols [45]. In addition, the high cost of these technologies and their accessibility by consumers need to be considered.

Natural based solutions may be interesting alternatives. Biomimicry, theorized in 1997 by Janine Benyus [46], is an area of research that draws on existing solutions in nature to respond to human needs. This approach has been used in architecture to build more energy-efficient buildings and to improve their autonomy, therefore reducing the environmental footprint on the urban metabolism. In this context, phytoremediation—using plants to remove toxins from air—was proposed in the 1970s as an efficient and cost-effective means to ameliorate the indoor air quality of NASA life-support systems [47]. Air pollutant amelioration by plants has been reported previously, and includes the ability of some species to absorb benzene from air [48]. In addition, a number of studies have reported that potted ornamental plants can remove VOCs from indoor air at different rates [49,50,51].

Although the use of plants as cleaners of indoor air is an attractive and cost-effective means to improve indoor air quality, the scientific data is not yet conclusive. Some studies [15,52,53] have highlighted the weak capacity of plants, by themselves, to improve indoor air quality at a full scale; to achieve this objective a high density of indoors plants would be necessary. Several challenges remain that require further investigation, such as understanding the mechanisms involved and the role of the constituents of the system (plant, soil, and microorganisms), in order to understand, optimize, and increase its efficiency.

Another innovative and promising possibility is the creation of buildings, and potentially cities, that are powered by microalgae. This approach would contribute to the development of more environmentally friendly and sustainable cities with greater biodiversity. In the following sections, the potential of using microalgae to clean indoor air, and particularly to recycle dirty indoor air, is analyzed in detail.

## 4. Microalgae for Treating Indoor Air

Microalgae are a varied group of photosynthetic unicellular microorganisms, with over 10,000 species, including blue algae/cyanophytes, protists, and other taxonomic groups [54]. They have few growth requirements, particularly in terms of nutrients needed, and can efficiently remove CO_2_ from the air or the waste streams of various origins. About 70% of the Earth’s atmospheric O_2_ has its origin in oceans, and microalgae are responsible for approximately 50% of this oxygen [55]. Microalgae are among the most efficient photosynthetic organisms for carbon capture and high biomass productivity [54,56]. Microalgae accumulate different compounds, such as lipids, fatty acids, pigments, polysaccharides, proteins, and carbohydrates, with different potential applications [57,58]. In addition, microalgae are a potential feedstock for achieving sustainable development goals [3] because they can be used to generate bioenergy and biofuel while contributing to carbon capture and utilization [59,60].

**Figure 1:**
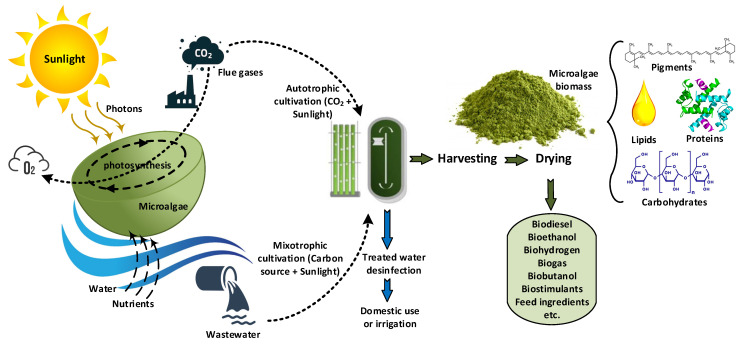
The functions of microalgae as solar energy collectors, CO_2_ biofixers, and energy storage as biomass through cultivation, and the potential applications of biomass components (authors’ own adaptation from [62]).

Microalgae growth relies on different factors such as: (a) light exposure: indirect radiation is usually preferred (1000–10,000 lux) because direct radiation may hinder efficiency (e.g., a lower CO_2_ biofixation rate of microalgae due to photo-inhibition); (b) adequate temperature range (16–27 °C for most microalgae); (c) CO_2_ supply through CO_2_-enriched air circulation; (d) stirring, to ensure that all microalgae cells are exposed to radiation and reduce sediments; and (e) nutrient availability [61]. Although a significant amount of research on microalgae cultivation and biomass processing is required, at the current state of development, microalgae are used at the industrial scale to obtain certain products or to perform certain tasks, such as carbon capture from waste streams or wastewater treatment (Figure 1).

In practice, microalgae can be used to remove contaminants from indoor air, or even outdoor air, by removing contaminants and CO_2_, rendering it more suitable for use in living spaces, thereby fulfilling air quality regulations. This is similar to carbon capture from flue gas, in which the CO_2_ and other contaminants are used as nutrients for microalgae growth [63,64]. In addition, when the outside air is particularly polluted, or cooling or heating is needed due to the variation in indoor or outdoor air temperatures, another efficient approach is to recirculate the air in a closed loop between the microalgae cultures and the living spaces, in which the microalgae system purifies the indoor air.

Therefore, motivated by the above-noted need to improve energy efficiency in public buildings, and thus promote sustainable systems, microalgae are promising microorganisms for multiple future applications, when applied in combination with imagination and inspiration [65]. One of the inspirations in this sense is to join microalgae systems to buildings to provide better indoor air quality, and thermal and aesthetic comfort. This can be achieved via the production of oxygen and fresh air for room ventilation, in a manner that is almost independent from the quality of the atmospheric air. The rationale for this arises from the fact that, in many European cities, atmospheric air does not meet the quality guidelines defined by the World Health Organization [23,66]. Official data provided by the Portuguese Environmental Agency for 2017 (the latest data available), recorded over 600 million kg of total CO_2_ emissions in the municipality of Porto, which is equivalent to a spatial concentration of 14.5 kg of CO_2_/m^2^ [67]. Due to this scenario, which appears unlikely to improve without intervention, there is a need to find alternative means to provide air in cities that is cleaner, and hence safer, than the surrounding atmospheric air. Therefore, in polluted environments, such as the case of certain cities, compliance with this legislation may increase the need for high air flows for renovation, with a corresponding sharp increase in the energy consumption parcel for ventilation (VC); however, there is no guarantee that safe indoor air would be provided. Furthermore, in highly polluted areas it can be difficult to achieve the clean air quality needed by people with respiratory problems, particularly in the most vulnerable groups, i.e., the elderly and children. Thus, the solution of integrating microalgae production systems in buildings potentially enables the quality of the indoor air to be better than that of the outdoor air.

**Figure 2:**
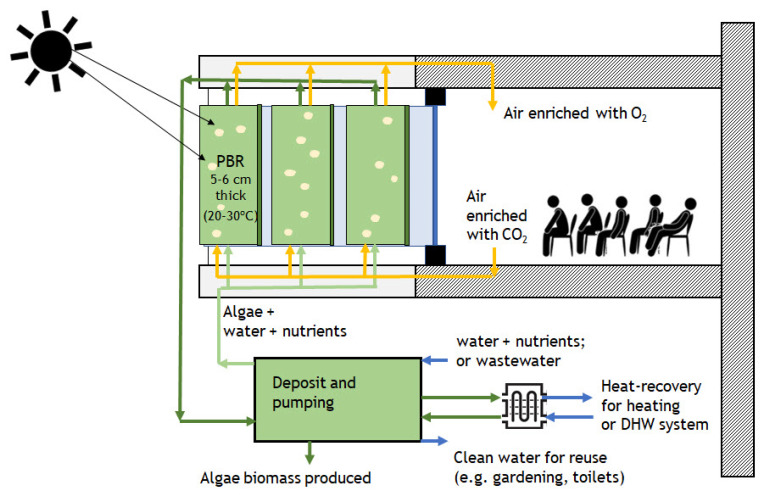
Microalgae cultivation system integrated into a building as the proposed solution for indoor air treatment, showing the air flow in and out of a room (authors’ own creation).

As shown in Figure 2, in the proposed system, air extracted from a room is directly injected into the microalgae cultivation photobioreactor (PBR) system, which provides a CO_2_ source for the cultures. In return, microalgae convert CO_2_ into O_2_ during their photosynthetic and metabolic activities. The produced O_2_, as a component of the aeration exhaust airflow from the PBR, is sent to the room. Airflow from the PBR is admitted into the room via a duct, thus using and transforming the current Heating, Ventilating, and Air Conditioning (HVAC) system.

As represented in Figure 2, exhaust air from rooms or other building spaces, such as classrooms, is admitted into the microalgae production system, where CO_2_ is converted into O_2_ and microalgae biomass is produced. Rich O_2_ air leaving the microalgae system is connected to air handling systems (AHS), rather than (or complementing) outdoor atmospheric air, to be filtered and corrected for temperature and humidity levels, and then admitted into the classrooms for ventilation purposes. In crowded closed spaces, such as classrooms, the CO_2_ exhaled by humans may reach levels higher than 5000 ppm which, although not presenting a risk to health, may affect the cognitive performance of the room’s occupants. Adequate ventilation procedures should be taken to avoid levels of CO_2_ concentration in indoor air that may pose risks to health [22].

Implementing microalgae systems in buildings is also considered to have significant future potential regarding the contribution of microalgae to the energy efficiency of buildings. One of the most significant forms of energy consumption in buildings refers to the HVAC system, which can be divided in two energy parcels: ventilation consumption (VC), regarding air renovation with the input of fresh air from outdoors; and thermal conditioning consumption (TC), regarding temperature regulation of the environment. However, because TC heavily depends on VC, as frequently reported in the literature, energy consumption reduction in an HVAC system is accomplished by reducing the VC parcel, which means the quantity of atmospheric air input is less than the necessary amount [32].

**Figure 3:**
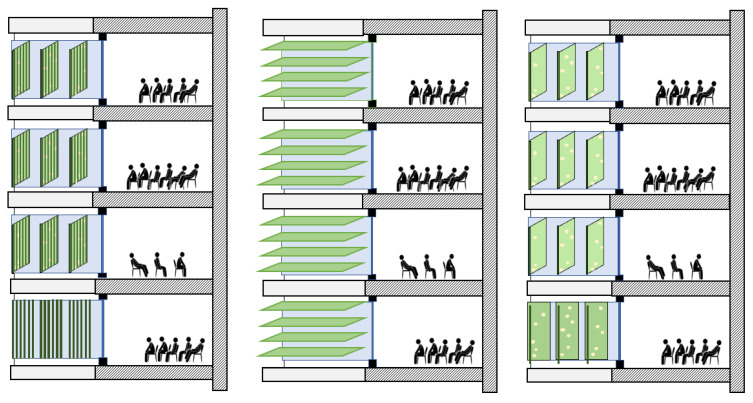
Examples of different assembly types of PBR panels on building façades that may be chosen according to the climate conditions at the building site. Different PBR panel orientations also result in different levels of light penetration and harvesting (authors’ own creation).

Thus, the association of microalgae with air handling systems (AHSs) can improve both the indoor air quality and thermal regulation, thus enhancing the building energy efficiency. As dense cultures, PBR systems can considerably decrease light penetration if mounted on the building façade (as shown in Figure 3), which has a direct effect on the building’s heat absorption. PBRs can also be integrated directly into a window, or easily mounted on the windows as biocurtains, through which, depending on the culture density, the light penetration can be adjusted. PBR panels can also be enabled to change their orientation on the façade according to the light direction. Examples of possible orientations of PBR panels on a facade are shown in Figure 3.

Thus, microalgae systems can be seen as examples of green vertical systems (GVSs). Alternatively, they can also be positioned as green horizontal systems (GHSs), such as in the case of green roofs. These green systems can be considered to be vertical gardens (in some cases involving vascular plants) integrated into buildings, which can be applied to both exterior and internal walls of a building. These systems have been increasingly considered by architects due to their various benefits at urban and building scales, despite their high initial costs [68]. Examples of these benefits include noise reduction; enhancement in urban air quality; improvement in energy efficiency; reduction in the urban heat island effect; improvement in the aesthetic of boroughs; value property increment; and, in particular, the improvement of the mental and physical health of citizens outside and inside the buildings [69].

Wong et al. [70] conducted a field experiment in Singapore showing that the application of green roofs contributes to the thermal benefits of both the buildings and their surrounding environments. Green roofs are an established technology in the construction sector. In addition, a growing number of stakeholders, both public and private, have recently become interested in green façades due to their potential energy, environmental, and human well-being benefits [71].

Some of these systems employ PBRs in the form of tubular or panel transparent vessels for the microalgae culture, which capture solar energy using microalgae photosynthetic activity, and thus produce biomass and biofuels [72,73]. Simultaneously, these systems help to reduce the buildings’ energy consumption, mainly due to thermal regulation [74]. The strategic implementation of microalgae production systems in buildings not only enables sunlight energy to be captured, but also to be stored in the form of microalgae biomass, which can be used for obtaining biofuels and valuable compounds with several potential applications (as shown in Figure 1) [75]. Microalgae PBRs mounted on a roof top or a façade produce oxygen through photosynthesis in the presence of sunlight. Microalgae can also obtain nutrients from wastewater generated in building, and capture the CO_2_ released by boilers, while supplying oxygen [76]. This means that the PBR system can be integrated into a building using a circular economy perspective, at least in terms of the valued resources, such as water, gases, and energy.

Additionally, the microalgae biomass generated can be used to produce biogas, which can be a source of heat and electricity, e.g., for providing hot water and heating for the building. This approach contributes to the status of a building as “low energy”, i.e., consuming less than 50 kWh m^−2^ year^−1^, as defined in the “Paris Climate and Energy Action Plan” [77]. Thus, a building can function as a green, renewable, and ecological factory, constituting a complex but circular system that recycles and regenerates its own needs. Such a building would not only provide shelter and comfort for its residents, but also be part of a group of similar buildings in a future sustainable city. From a biorefinery perspective, regarding the potential applications of microalgae biomass, these building systems could be part of a local supply chain for food supplements, pigments, and biofuels. In addition, the use of microalgae as a potential source of renewable energy (biodiesel, bioethanol, biohydrogen, or biogas) for use in the building itself would be an important contribution to sustainable and self-sufficient construction [78,79].

Concerning the production of biogas from microalgae, previous research [80] noted that it is a highly challenging substrate for anaerobic digestion due to the microalgae’s cell wall recalcitrance and high protein content. This is unfavorable for fermentation, and requires additional pretreatment and co-fermentation strategies for the process to be sufficient. However, intensive recent research in this area revealed that it is possible to obtain microalgae biomass with low protein content by cultivating microalgae in a nitrogen-deprived medium. A previous study [80] showed that the anaerobic digestion of microalgae biomass with low protein content resulted in a stable process with low levels of inhibitory substances, a high biogas yield, and high methane productivity, corresponding to a biomass-to-methane energy conversion efficiency of up to 84%. This made it possible to generate biofuel in a sustainable and low-cost manner, while avoiding the need for biomass pre-treatments and the intensive use of energy. Thus, the process was found to be feasible and enabled positive net energy to be obtained.

In the building construction sector, in which costs are a critical factor, the potential economic viability of the PBR system based on the use and valorization of the produced microalgae biomass (e.g., through the extraction of pigments with a high market value or renewable energy generation) may be the decisive factor for the efficient implementation of these systems in buildings [81,82,83]. Branco-Vieira et al. [84] showed that the items with the greatest contribution to the final cost of producing microalgae biomass in closed PBRs are the capital costs (53%), followed by labor (25%) and electricity (11%). Fertilizers contribute less than 1% to the biomass production cost. These authors also showed that the annual operating costs for microalgae biomass production (including water, electricity, labor, fertilizer, and wastewater treatment) represent about 55% of the initial capital investment in closed PBR systems (including PBR construction, circulation pump, heating and cooling equipment, centrifuge, process control, and infrastructure).

Pilot projects are currently underway, but little information is available about their performance, both as energy providers and as a full ecosystem [74]. In 2013, the BIQ House (building with Bio-Intelligent Quotient) was constructed in Hamburg, Germany, as a low-energy residential building [85]. The building includes 120 flat panel glass photobioreactors mounted on the façade (the so-called “SolarLeaf” façade), covering an area of 200 m². The flat PBRs used on this building were claimed to be highly efficient for microalgal growth and to require minimal maintenance. The implemented system provides about one-third of the building’ total heat demand for the 15 residential units, and has the capacity to generate biomass and heat as sources of renewable energy. Additionally, the PBR system provides a thermally controlled microclimate around the building, providing noise reduction and dynamic shading. This PBR system has the capacity to remove up to six tons of carbon dioxide per year, using the flue gas from the gas burner as the CO_2_ source to produce microalgae biomass. Using an external production unit, up to 80% of the harvested biomass is converted into methane, which is then returned to the building to generate electricity and heat [86].

Araji and Shahid [87] suggested the integration of PBR systems in façades, using different combinations of flat-plate microalgae PBRs and glazing panels, depending on the building design and panel orientations. The authors analyzed the contribution of microalgae to energy generation, the capacity for CO_2_ biofixation, and the land preservation, considering two possible microalgae species: *Chlorella vulgaris* and *Dunaliella tertiolecta*. Results showed that the use of the former species with a panel inclination of 90° sequestered 89% more CO_2_ than *Dunaliella tertiolecta* at a panel inclination of 75°.

Negev et al. [88] also considered two microalgae species—*Chlamydomonas reinhardtii* and *Chlorella vulgaris*—to analyze the potential energy benefits (energy consumption, window U-value, visible transmittance, and solar heat gains) of incorporating PBR systems into the window façades of an office building in Tel-Aviv, Israel. Compared with single glazing, the energy savings of the PBR window solution varied widely with the façade orientation and the microalgae biomass concentration inside the PBR system. With the maximum microalgae concentration, the saved energy varied from 20 kWh m^−2^ year^−1^ on the south façade to 8 kWh m^−2^ year^−1^ on the east façade, whereas energy consumption increased on the north façade by 18 kWh m^−2^ year^−1^. This study noted that a simple incorporation of a microalgae PBR into the windows was able to improve building thermal performance under specific conditions. The conditions in this study were examined using thermal simulation, based on adequate window sizing and orientation in a Mediterranean climate context. Results showed the building’s HVAC loads were reduced. As with other technologies, to take maximum advantage of microalgae technology, and to improve a building’s energy efficiency, an appropriate building design is necessary [89]. In addition, because of the use of roof tops or façades, PBR solutions for growing microalgae avoid the impact of land use change, and the microalgae biomass can be used for other applications (e.g., biofuel production or biomaterials).

Biloria and Thakkar [61] analyzed the economic and environmental performance of two alternative renewable energy sources—microalgae building technology and photovoltaic (PV) panels—used to retrofit the front façade of a multistory building in Sydney. The microalgae closed PBR system comprised a set of 0.1 m diameter tubes, separated from each other by 0.05 m, and located 0.5 m from the façade. The tubes were connected to an anaerobic digester, which was placed in the basement, to produce biogas from the microalgae biomass. Wastewater from the building was also pumped to this digester for purification by the removal of pathogens. For a PBR area of 1500 m^2^, the system was expected to produce 28.5 kg/day of microalgae biomass, with an energy demand of 2.4 kWh m^−3^day^−1^. Thus, this microalgal PBR system could be integrated with the other functions in the building. During their growth, the microalgae biofixed carbon, and the produced biomass was able to be used to generate bioenergy (e.g., cogeneration of electricity and heat from biogas obtained by microalgae biomass via anaerobic digestion) or as fertilizer for agriculture. Microalgae were also able to be used to treat the wastewater generated in the building, using the chemical and biological content of the wastewater as nutrients for their growth. Thus, this water was recycled (e.g., for flushing toilets or for irrigation of the surrounding gardens), reducing the building annual water consumption by 25% (13,704 m^3^). In this study, the PV panels were identified as being more feasible (with a shorter payback time) than the PBR system, which would only become economically preferable to the PV system after a useful lifetime of over 36 years. Nevertheless, the authors concluded that the microalgae technology (closed tubular photobioreactors) had more environmental benefits than the solar PVs, given the local challenge of addressing water scarcity, and reducing air pollution and carbon emissions.

Pagliolico et al. [90] evaluated the optical performance of disposable plastic bags used as circular cubicles for microalgae production, such as in the form of shading systems or static screens for windows. The authors concluded that the microalgae system resulted in an increased daylight level and glaze in a room, compared to glazing with Venetian blinds, which resulted in lower energy demand (by up to 57%) for lighting.

**Figure 4:**
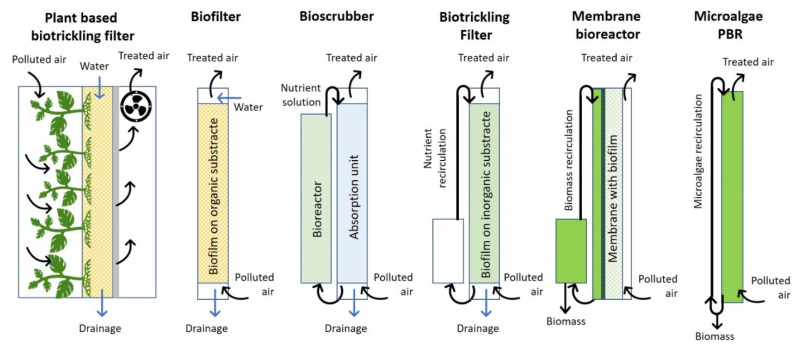
Scheme of different biotechnologies for air purification as suggested by González-Martín et al. [15] (authors’ own adaptation from the source).

Different biotechnological strategies can be used to improve indoor air quality. One such approach is living wall systems (LWSs). These are vertical hydroponic systems that can also function as biofilters. An LWS supports vegetation that is rooted in walls or in a substrate attached to the wall [52]. The LWS can also function as a bioreactor for the cultivation of microorganisms, such as microalgae. González-Martín et al. [15] reviewed and highlighted the potential applications of indoor air pollution mitigation strategies, in addition to technological solutions, used to improve indoor air quality, including mechanical, chemical, and biological purification systems. Examples of biotechnological strategies for indoor air purification that can be engineered in multiple configurations, with their principal operational characteristics, are presented in Figure 4 [15].

As suggested by González-Martín et al. [15], the active biotechnological systems shown in Figure 4, such as bioscrubbers, biotrickling, biofilters, membrane bioreactors, and microalgae photobioreactors, can more effectively remove VOCs and PM from indoor environments than passive systems, such as indoor plant pots. Because passive systems depend on the diffusion of polluting gases, they usually operate slowly and at lower concentrations. In contrast, active systems increase the availability of polluting gases by incorporating mechanical ventilation devices; thus, air-cleaning rates may be significantly higher [52]. In addition to air purification, these botanical systems also have other benefits, due to plants’ evapotranspiration, including reduction of the temperature around the plants. Plants can help to cool the air and control humidity (an air moisture content in the range of 30–70% is generally recommended for comfort). Additionally, they can act as an acoustic insulator [52]. In plant-based biotrickling filters, the polluted air is forced to flow through the aerial parts and roots of hydroponic plants, which are fixed on an inert material (e.g., ceramics or plastic resins). A nutrient solution is continuously trickled down over the packing, thus maximizing the removal of pollutants. In bioscrubbers, the air is forced through an aqueous phase, to which the air pollutants are transferred, thus cleaning the air. Then, the aqueous phase is transferred to a bioreactor in which the pollutants are biodegraded. In biofilters, polluted air passes through a porous, moist material that supports microbial growth; this is commonly an organic material such as compost. The growth medium is generally a natural material that is biodegradable and provides the nutrients necessary for the microorganisms’ growth. Membrane bioreactors can also be used to remove VOCs at high concentrations in the air [15,52]. Most of these biofiltration technologies are well established and have proven themselves in industrial applications for air pollution control. These systems have demonstrated good performance and reliability, and low operating costs; for example, removal efficiencies greater than 95% in residences in times of less than 95 s were achieved for a wide range of pollutants [15].

## 5. Critical Analysis of Microalgae Indoor Purification Systems

Despite the considerable costs, the effective integration of microalgae PBRs systems into buildings may have the following advantages [75,91,92,93]:

- Improvement of air quality—through photosynthesis, the CO_2_ from indoor air, produced by the building’s occupants, is used to produce O_2_ by microalgae cells. During the exchange of the indoor air (CO_2_) with the O_2_, the microalgae in the PBR act as a biofilter of the air, contributing to improve its quality.
- Thermal comfort—the microalgae PBR panel acts as a biocurtain, blocking or reflecting the light. In particular, the dense microalgae cultures provide greater blockage of light.
- Aesthetic design—a green façade makes a building more attractive.
- Low environmental impact, avoiding the intensive use of mineral resources that is required with solar panels or batteries.
- PBRs mounted on façades do not require additional land use.
- These systems lead to a reduction in greenhouse gas emissions, in particular, carbon, because microalgae absorb CO_2_ during photosynthesis while producing oxygen.
- Because microalgae absorb sunlight, bioreactors on façades act as dynamic shading devices for the building.
- Microalgae can be fed with the necessary carbon taken from a nearby generation process, or even grey water generated during the building’s normal operation.
- Microalgae biomass can be used flexibly for power and heat generation.
- Possible revenue from biomass sales may offset energy costs.
- From a biorefinery perspective, innovation and the development of new energy sources from biomass and/or sources of renewable raw materials enable other products and compounds to be obtained.
- Potential integration with wastewater processing systems at a local scale. This is relevant in isolated buildings or in areas in which buildings are highly dispersed.
- If implemented at a larger scale, these systems may have the potential to mitigate the effect of the urban heat island.

Therefore, buildings equipped with microalgae systems for indoor air quality improvement can have positive social, environmental, and economic impacts. Microalgae systems can be considered to be living laboratories. Their use is expected to provide important insights to enhance knowledge regarding cities’ sustainability and the potential responses of cities to mitigate and adapt to the challenges of climate change. According to the World Health Organization (WHO), populations do not breathe safe air in 49% of cities of high-income countries (including cities in Portugal), that is, atmospheric air does not meet WHO Air Quality Guidelines [23]. Poor outdoor (atmospheric) air quality influences the indoor air quality of buildings, even if proper air handling systems (AHSs) are installed.

Microalgae systems provide numerous environmental benefits. Nonetheless, valid, although not insurmountable, concerns need to be addressed that may constitute barriers to the development and adoption of these systems [87,91]:

- The high costs of new technologies compared to already established commercial technologies;
- PBR systems include several glass panels and tubes with valves that require maintenance (cleaning and periodic replacement), which may be onerous;
- Maintenance requires training and education of professionals to ensure continuous optimal performance;
- With new technologies and innovations, there is a risk that they will not work as planned;
- The PBR system may have to be shut down in the winter, due to the lack of sunlight for photosynthesis and, during the hot months there may be the problem of overheating;
- Better control systems for the temperature and hydrodynamic stress in flat-plate PBRs still need to be developed;
- Concerns about the total carbon and water footprints associated with the entire life cycle of the PBR systems coupled to a building should be addressed;
- The possibility of contamination of microalgae cultures;
- The possibility of losses and leaks that also could cause odors;
- Some microalgae contain toxins that are harmful to human health; this demands a rigorous quality control of the cultivation conditions;
- It is necessary to assess whether other renewable energies, such as solar thermal, photovoltaic, and wind energy, are able to produce more energy than the microalgae biomass, depending on the production rates or sunlight periods throughout a year;
- It is still not clear which microalgae species, or consortia of microalgae, with or without other species of microorganisms, will be more suitable depending on the local climatic conditions and concentration of CO_2_ and/or other pollutants, among other factors.

To minimize the economic risks of these microalgae production systems, it is necessary to evaluate their performance at the proof-of-concept and pilot scales before implementation. Life cycle analysis and techno-economic evaluations also need to be undertaken earlier in the conceptual design stage to identify the most viable options [94,95]. The identified optimal alternative can then be prototyped and assembled at a pilot scale with the aim of developing more sustainable processes. As a result, more detailed and objective designs of real-life systems can be developed and implemented in practice.

## 6. Conclusions

The issue of indoor air quality has gained greater visibility due to the COVID-19 pandemic. In airborne diseases, such as COVID-19, the air is the medium by which viruses are transported and transmitted between people, in the form of aerosols and fine particles. As a result, it is well known that indoor spaces must be ventilated, and that indoor air must be treated, to prevent infection. The ventilation of indoor spaces is a common procedure used in buildings to provide thermal comfort and high-quality indoor air. This is traditionally undertaken by recycling most of the indoor air to prevent an additional energy load in the heating or cooling of the atmospheric air from outdoors. By comparison, new strategies involve the elimination of recirculation and the intensification of filtration. Both of these processes entail a higher energy demand and, consequently, higher costs, at the financial and environmental levels. Thus, it is important to consider new alternatives to ensure healthy indoor air at a reduced cost and a high energy efficiency. This article aims to highlight the potential of using microalgae systems to clean indoor air in buildings, based on the circular economy concept of reducing the use of resources and maximizing their benefits. The presented literature review discusses the related previous research, and the advantages and limitations of these microalgae systems are identified. It can be hypothesized that buildings equipped with microalgae systems may improve positive social, environmental, and economic impacts. Microalgae “living labs” are expected to provide important knowledge regarding the responses of cities and the specific solutions to address the challenges of climate change. More studies are necessary to refine these systems and overcome the existing problems.

Cities and buildings require combined and diversified efforts to design tailored solutions that are suitable for their specific needs. A microalgae PBR system is a promising alternative for improving the quality of indoor air. However, for practical and economic reasons, the design of the PBR system must be economically viable, particularly when compared with existing indoor air purifications systems. Such a comparison must consider the ease of maintenance, cleaning, and operation. In addition, the PBR system must be built with lightweight and durable materials to enable it to withstand the external environment. Furthermore, the optimal microalgae species or consortia still need to be specified, according to the local climatic and application-specific conditions. These aspects should be addressed in future R&D programs and projects, to further highlight the potential of microalgae-based systems for indoor air purification.

## Figures and Tables

**Table 1:** Some indoor air pollutants, their sources, and their effects on human health.

Agent	Health Effects	Sources
Benzene	Carcinogenic and mutagenic	Indoor: Combustion processes, such as cooking, candles, incense, fireplace, and smoking. Outdoor: Traffic, industrial pollution, etc.
Toluene	Suspected to be reprotoxic, effects on central nervous system, skin irritation	Mainly indoors: Combustion processes, building materials, do-it-yourself activities (e.g., painting and gluing), printing, photocopying, etc.
Styrene	Suspected to be reprotoxic, effects on central nervous system, skin and eye irritation	Mainly indoors: Building materials (PVC—polyvinyl chloride, insulation materials), paints, consumer products, etc.
Tetrachloroethylene	Suspected to be carcinogenic	Mainly indoors: Dry cleaning textiles, stain removers, water repellents, wood cleaners, etc.
Formaldehyde	Carcinogenic, suspected to be mutagenic and skin sensitizing	Mainly indoors: Building materials, furniture, combustion processes (incense, fireplace, smoking), etc.
Dibutyl phthalate	Reprotoxic and recognized in the EU as an endocrine disruptor	Mainly indoors: Used in the plastics part of building materials and consumer products, etc.
Nitrogen dioxide	Respiratory problems	Indoor: Combustion processes, such as cooking, candles, incense, fireplace, smoking, etc. Outdoor: Traffic, industrial pollution
Carbon monoxide	Toxic by inhalation, provokes hypoxemia	Indoor: Combustion processes, such as cooking, candles, incense, fireplace, smoking, etc. Outdoor: Traffic
